# Coping Style and Quality of Life in Elderly Patients with Vision Disturbances

**DOI:** 10.1155/2014/584627

**Published:** 2014-08-21

**Authors:** Maria Oles, Piotr Oles

**Affiliations:** Institute of Psychology, John Paul II Catholic University of Lublin, Aleje Raclawickie 14, 20-950 Lublin, Poland

## Abstract

*Purpose.* This study aims at evaluating coping style and quality of life in patients with glaucoma and cataract. *Methods.* The participants were patients (*N* = 237, 130F; mean age: M = 67,8; SD = 9,5) with low vision caused by cataract (*N* = 188) and glaucoma (*N* = 49) who answered the Quality of Life Questionnaire (QOLQ) by Schalock and Keith. The participants were divided by means of cluster analysis (*k*-means) according to coping styles measured by CISS (Endler and Parker) into three groups: (1) high mobilization for coping, (2) task-oriented coping, and (3) low mobilization for coping. *Results.* In all the group, a general quality of life was moderately lowered; however, in task-oriented group it was relatively high. Moreover, task-oriented group had significantly lower level of anxiety (STAI), hopelessness (HS), and loneliness (UCLA LS-R) and higher level of self-esteem (SES) in comparison to the patients from high mobilization and low mobilization for coping. *Conclusions.* In an old age, adaptive coping with vision disturbances does not necessarily mean flexibility in combining all coping styles, but rather task-oriented coping and an ability to use social support. Extreme mobilization for coping seems not adaptive similarly like low mobilization for coping because it violates balance between environmental requirements and personal resources.

## 1. Introduction

Vision serves as a vital function ensuring the feeling of control over the immediate surroundings and creating a sense of contact with the world. Therefore, the fact does not render it peculiar that cases connected with vision disturbances and eyesight loss entail heightened anxiety and the fear for autonomy limitation [1–3]. Statistical data prove that the risk of developing cataract, glaucoma, and other ophthalmological disorders increases with age [4, 5]. Cataract affects almost 20% of people between the ages of 65 and 74 and for 50% of those who are between 75 and 84 years of age becomes the main factor leading to blindness [6, 7]. The risk of developing glaucoma, on the other hand, occurs in 2% population segment of forty-five-year-olds and older patients, increasing markedly in the case of people at an advanced age. Due to the fact that initial stages of the disease development are difficult to diagnose and since the diagnosis itself may lack precision, a strong feeling of anxiety can arise [8–10]. Low vision is one of the most important reasons of lowered quality of life, anxiety, and poor adaptation, especially in older patients who have two kinds of problems, relating to their health and age [11–13]. Thus, coping with stress seems especially important for adaptation to low vision [14].

The research projects on the patient groups focus predominantly on establishing the quality of life pattern and on examining changes both in the paradigm and in the vision-related functional status after the treatment. Only a fraction of these researches dealt with such variables as depression and anxiety, expectations concerning the future, the feeling of loneliness, or self-esteem [15, 16]. The importance of psychological variables such as strategies of coping or anxiety intensification factor has been duly underlined in glaucoma research due to the fact that the concealed development of the disease and the possibility of unfavourable prognosis may potentially lead to the development of the feeling of anxiety [8, 17]. According to current research results both socioeconomic factors and psychological variables influence quality of life in elderly patients with vision disturbances [2, 18–20].

This study has been intended to constitute a probe into coping styles in cases of patients with vision disturbances to demonstrate interrelationships existing between the emerging pattern and quality of life assessment in cataract- and glaucoma-affected patients. The problem can be formulated in the following questions.

1. What styles of coping do patients with vision disturbances adopt?
2. What is the adaptive value of the styles of coping exhibited by elderly patients with vision disturbances who undergo cataract and glaucoma treatment?

Quality of life is defined as “an individuals' perception of their position in life in the context of the culture and value systems in which they live and in relation to their goals, expectations, standards, and concerns” [21, p. 1405]. Quality of life evaluation was employed to designate the psychic adaptability coefficient. Additionally, the levels of self-esteem and such clinical variables as anxiety, depression, and loneliness were also diagnosed. The project was aimed at having a predominantly exploratory character. Its goal was to detect the styles of coping most frequently assumed in this group of patients and to test their adaptive value. In light of the literature on the subject, task-oriented coping and adaptive flexibility figure as having adaptive value. Therefore, it was postulated that H1: moderate mobilization of different styles of coping showing clear tendency towards employing task-oriented coping has adaptive value and that H2: coping based on emotion-oriented and avoidance-oriented coping has lesser adaptive value indicated by relatively low quality of life, low self-esteem, and a heightened level of anxiety, hopelessness, and loneliness.

## 2. Sample and Methods

The sample consisted of 237 patients (mean age: M = 67,8; SD = 9,5) including 188 cataract and 49 glaucoma patients. A time frame for the recruitment of the participants was one year, and they were consecutive patients, except those who refused to participate in this investigation due to their poor well-being or lack of motivation. Since there were no major differences regarding quality of life or coping styles, both glaucoma patients and cataract patients were treated as a unit irrespective of their sex.

Coping styles were investigated by means of the Coping Inventory for Stressful Situations (CISS) by Endler and Parker [22]. The inventory consists of three main scales: task-oriented coping (TOC), emotion-oriented coping (EOC), and avoidance-oriented coping (AOC), with the last one with two subscales: engagement in substitute activity (ESA) and seeking for social relationships (SSR). Cronbach alpha for three main scales checked in a sample of elderly patients with low vision (*N* = 50, 25 male and 25 female) is 0.81, 0.78, and 0.74, respectively, and for subscales it is 0.60 and 0.67.

Quality of life was measured with the help of The Quality of Life Questionnaire by Schalock and Keith [23]. The questionnaire consists of 40 items measuring the quality of life in four domains, each comprising 10 items: satisfaction, competence/productivity, empowerment/independence, and social belonging/community integration. The fifth domain was added to the present research: health related quality of life (5 items). The questionnaire is based on an interview containing 45 questions, each with 3 possible answers scored 1, 2, or 3—from low to high quality of life. Reliability of the Quality of Life Questionnaire (40 items), checked in cataract sample (*N* = 50, 25F and 25M), is Cronbach alpha = 0.86 and for each scale 0.81, 0.86, 0.58, 0.50, and 0.79, respectively.

The following scales were used to assess other variables indicating psychological functioning of the patients: Self-Esteem Scale (SES) by Rosenberg [24], State-Trait Anxiety Inventory (STAI) by Spielberger and Reheiser [25], Hopelessness Scale (HS) by Beck et al. [26], and Loneliness Scale Revised (UCLA LS-R) by Russell et al. [27].

## 3. Results and Discussion

In order to isolate subgroups of patients employing distinct styles of coping, cluster analysis based on *k*-means method and conducted on the standardized scores in three main scales of CISS by Endler and Parker was carried out. Three groups of patients exhibiting different coping styles were thus singled out:

1. patients exhibiting high mobilization for coping with emotion-oriented and avoidance-oriented coping prevalent (*N* = 89, including 68 cataract patients and 21 glaucoma subjects);
2. patients exhibiting task-oriented coping (*N* = 69, including 59 cataract patients and 10 glaucoma subjects);
3. patients exhibiting low mobilization for coping (*N* = 79, including 61 cataract patients and 18 glaucoma subjects).

According to CISS scales, all of the differences emerging between the groups were significant (*P* < 0.001) (cf.  Table 1). There are no major discrepancies in medical variables describing acuity and quality of vision.

Interestingly enough in all three groups three main coping strategies were used to a lesser or higher degree. In the first group, avoidance-oriented coping and emotion-oriented coping appear to be concurrent with each other. Furthermore, the patients belonging to this group achieved comparatively high scores as far as task-oriented coping is concerned, and this suggests flexibility of coping. The second group demonstrated task-oriented coping combined with the inclination to search for and employ social support. In the third group, the level of mobilization to cope was relatively low. The division of thus delineated groups with respect to quality of life revealed several crucial differences (Table 2).

The second group, namely, patients characterized by task-oriented coping, evidently reveals higher quality of life in contrast to the other two groups. This fact pertains not only to the overall result achieved on the Total Quality of Life Scale, but also to the results obtained in other two domains, that is, empowerment/independence and social belonging/community integration. It is also worth noting that, as for variables describing quality of life, there were no significant differences between the first group of patients with high mobilization for coping and the third group of patients with low mobilization for coping.

Further analysis aims at exploring the adaptive value of the styles of coping, respectively. On the basis of all the results presented so far, it transpires that task orientation can hold a higher adaptive value than the generally heightened as well as decreased mobilization for coping. Such an interpretation is plausible in light of higher quality of life in the majority of task-oriented patients in contrast to subjects belonging to the remaining two groups.

But why is it the case? In order to answer the question, the levels of anxiety (measured by means of State-Trait Anxiety Inventory (STAI)), of hopelessness (Hopelessness Scale (HS)), of loneliness (UCLA Loneliness Scale Revised (LS-R)), and of self-esteem (Self-Esteem Scale (SES)) were compared in the three selected groups. The juxtaposition of mean results in the scales measuring aforementioned clinical variables indicated that anxiety, hopelessness, and loneliness levels were markedly lower in the group of patients using mainly task-oriented coping (Table 3). From a statistical point of view, there existed no significant differences between the first group (with high mobilization and emotion-avoidance orientation) and the third group (with low mobilization for coping). For example, intensity of hopelessness (HS) reached a clinical level in both of the groups.

The level of self-esteem was higher in the group of task-oriented patients in comparison with the two remaining groups: with high and low mobilization for coping (there was no significant difference between the mean results in both groups). Both high and low mobilization for coping concurred with lowered self-esteem. Participants with high and low mobilization for coping had lower self-esteem than task-oriented patients and higher level of clinical variables such as anxiety, hopelessness, and loneliness. This implies that high mobilization for coping was not necessarily an adaptive orientation for patients with vision disturbances as well as the absence of mobilization in confrontation with stress. Research results seem to emphasize the fact that not only mobilization embodying all the styles of coping—which implies flexibility—has adaptive value. For adaptive functioning, two factors appear to be decisive: the effort put into the process of coping and the apt guiding of the process towards task-oriented coping. Moreover, the tendency towards searching for social support was also exhibited and it could be interpreted as task-oriented in the case of elderly persons with visual disturbances—to keep and develop social support seems a big challenge for the elderly and the sick [28].

The conducted studies once again attested to the adaptive value of task-oriented coping and in general an active life in old age [19, 29, 30]. Patients adhering to this style exhibit higher quality of life and a lower level of anxiety, pessimism, and loneliness unlike subjects adhering to coping style based on emotions or avoidance. Furthermore, it was discovered that, to a certain extent, task-oriented coping entails searching for and employing social support, which, on account of the patient age and vision disturbances involved, appears to show adaptive value [29, 31, 32]. This conclusion together with the interrelation between coping and its adaptive value, on the one hand, and self-evaluation, on the other hand, is not characteristic of persons with vision disturbances exclusively. Similar results were achieved in other groups of patients [33, 34].

However, the result suggesting that both high mobilization for coping and the absence of mobilization seem to be equally nonadaptive becomes distinctive for the examined group of patients. The former case is often accompanied by noneffective exploitation of personal resources and the latter by passivity and the adoption of a resigned attitude which can be moderated by negative affectivity or depression [16, 35]; low quality of life cooccurring with an increasing level of anxiety, pessimism, loneliness, and lowered self-esteem is often generated in either of the cases [36]. Most probably, not so much the effort engaged as coping directed towards the task-oriented style and pertinent mobilization of the support of close persons, appropriate groups, and relevant institutions—which accords with positive self-evaluation and self-esteem—proves decisive in the process of adaptation throughout illness [17]. The ability of engaging oneself in substitute activities in place of disintegrated routine tasks appears to be equally important (e.g., concentrating on listening to the radio rather than watching television, involvement in gardening instead of reading papers, or going to the cinema).

Whenever the psychological corollary of disease symptoms developed due to cognitive functional disturbances—especially those related to visual perception—results in the loss of self-confidence, deterioration of social relations, poor performance of social or professional roles, the decline in the family status, and the development of negative emotions such as anxiety, tension, or lower self-esteem, the effectiveness of coping turns out to be vitally important [3, 15–17, 37–39]. However not all studies support the conclusion that visual impairment is necessarily linked to well-being or depression [40].

One should remember also about individual differences not only in effectiveness of given coping style but also in temperament [40] as well as in defining life domains which are important to the patients and which constitute their quality of life [41, 42]. At the same time, adaptive patterns in elderly persons may be affected by flexibility limitations. When faced with tense situations, they cope employing whatever methods are available or simply give up. The ability to retain self-esteem favours strengthening task-oriented coping and the ability to take advantage of social support, which, on its part, is conducive to lowering the anxiety, depression, and loneliness threshold.

There are some limitations of this study; first, this study, which is typical for questionnaire approach, based on self report data (and not verified, e.g., by objective observation), second, the research was conducted in one country (Poland), and, third, socioeconomic status of elderly people (rather low in most cases) could influence the results, especially assessment of quality of life, depression (i.e., hopelessness), or anxiety and loneliness.

While we have distinguished a few groups of patients different in coping with stress related to their illnesses, one can conduct similar analysis taking temperamental features as a criterion for division of the groups, providing also a relationship between coping, quality of life, and temperament. In fact, empirical data suggest that patients differ also in their psychological functioning due to specific temperamental features [40]. Moreover, temperamental factors can underline different coping strategies. It seems to be an interesting topic for further research, especially when such a study would be focused on how the patients face the illness as well as on more practical issue, namely, how to organize and propose better care for elderly people with vision disturbances.

## 4. Conclusions

The conclusions to be drawn from the presented study are as follows.

1. Patients who follow the pattern of task-oriented coping and who take advantage of social support are characterized by higher quality of life, higher self-esteem, and comparatively lower level of anxiety, pessimism, and loneliness. This proves task-oriented coping to be of greater adaptive value than passivity in stress exposure situations and a generally heightened mobilization for coping in emotion-avoidance pattern, in particular.
2. The results hint at the significance of social support and at the value of interpersonal relationships for quality of life in elderly persons with vision disturbances such as glaucoma or cataract, provided that task orientation is involved. The tendency towards searching for interpersonal interactions as a way of coping detected in the task-oriented group of patients can be plausibly accounted for. In the case of elderly persons coping with their illnesses, seeking and employing the support of other people are not exclusively of adaptive value, but they also appear to constitute a definite task enabling them to function on a daily basis.
3. Both demobilization and high mobilization for coping appear to be nonadaptive styles of functioning which entail lowered quality of life, lowered self-esteem, and increased level of pessimism, loneliness, and anxiety.

## Figures and Tables

**Table 1:** Coping styles in three groups of patients: comparison.

Group/CISS scales	Group 1 (*N* = 89)		Group 2 (*N* = 69)		Group 3 (*N* = 79)		Significant differences	
	M	SD	M	SD	M	SD	*F*(3,234)	*P* <
TOC	59,69	5,82	62,09	6,05	47,59	5,79	134,88	0,0001
EOC	50,98	6,66	34,57	6,14	42,11	7,09	119,68	0,0001
AOC	43,92	7,17	35,06	6,33	33,63	7,52	52,38	0,0001
ESA	15,76	4,44	11,65	3,27	12,18	3,63	27,65	0,0001
SSR	17,71	3,81	15,19	3,74	12,92	3,68	34,24	0,0001

TOC: task-oriented coping, EOC: emotion-oriented coping, AOC: avoidance-oriented coping, ESA: engagement in substitute activity, and SSR: seeking for social relationships.

**Table 2:** Quality of life in three groups: comparison.

Group/scale	Group 1 (*N* = 89)		Group 2 (*N* = 69)		Group 3 (*N* = 79)		Significance of differences	
	M	SD	M	SD	M	SD	*F*(3,234)	*P* <
QOL-TOT.	82,21	8,54	86,74	7,91	81,51	7,73	8,98^ab^	0,001
SATISF.	21,18	3,51	22,71	2,85	21,87	3,52	4,10^a^	0,05
COMPET.	13,61	3,85	13,77	4,70	12,51	2,86	2,49	—
INDEPEN.	26,38	2,41	27,43	2,00	25,99	2,93	6,57^ab^	0,01
INTEGR.	21,49	3,25	22,83	2,94	21,10	2,96	6,32^ab^	0,01
HRQOL	8,65	2,05	8,93	2,50	8,67	2,26	0,34	—

^a^Significant difference between groups 1 and 2, *P* < 0,05.

^b^Significant difference between groups 2 and 3, *P* < 0,05.

**Table 3:** Clinical and social-cognitive variables in three groups. Comparison: state anxiety (X-1) and trait anxiety (X-2), hopelessness (HS), loneliness (UCLA LS-R), and self-esteem (SES).

Group/scale	Group 1		Group 2		Group 3		Significance of differences	
	M	SD	M	SD	M	SD	*F*(3,234)	*P* <
STAI: X-1	43,16	12,70	33,90	10,27	42,49	11,32	14,66^ab^	0,001
STAI: X-2	45,45	8,98	36,47	6,07	43,99	8,90	25,61^ab^	0,001
HS	9,07	4,63	6,16	4,46	9,25	5,19	9,64^ab^	0,001
LS	35,76	8,94	30,44	7,30	36,53	9,76	10,26^ab^	0,001
SES	27,38	3,08	30,45	3,46	27,41	3,94	16,82^ab^	0,001

^a^Significant difference between groups 1 and 2, *P* < 0,05.

^b^Significant difference between groups 2 and 3, *P* < 0,05.
